# *In vivo* Neutralization of Pro-inflammatory Cytokines During Secondary *Streptococcus pneumoniae* Infection Post Influenza A Virus Infection

**DOI:** 10.3389/fimmu.2019.01864

**Published:** 2019-08-14

**Authors:** Niharika Sharma-Chawla, Sabine Stegemann-Koniszewski, Henrike Christen, Julia D. Boehme, Olivia Kershaw, Jens Schreiber, Carlos A. Guzmán, Dunja Bruder, Esteban A. Hernandez-Vargas

**Affiliations:** ^1^Frankfurt Institute for Advanced Studies, Frankfurt am Main, Germany; ^2^Immune Regulation Group, Helmholtz Centre for Infection Research (HZI), Braunschweig, Germany; ^3^Infection Immunology Group, Institute of Medical Microbiology, Infection Prevention and Control, Health Immunology, Infectiology and Inflammation, Otto-von-Guericke University Magdeburg, Magdeburg, Germany; ^4^Experimental Pneumology, University Hospital of Pneumology, Health Campus Immunology, Infectiology and Inflammation, Otto-von-Guericke University Magdeburg, Magdeburg, Germany; ^5^Department of Veterinary Medicine, Institute of Veterinary Pathology, Free University Berlin, Berlin, Germany; ^6^Department of Vaccinology and Applied Microbiology, Helmholtz Centre for Infection Research (HZI), Braunschweig, Germany; ^7^Centre for Individualized Infection Medicine (CiiM), Hanover, Germany

**Keywords:** co-infection, mathematical modeling, IFN-γ neutralization, IL-6 neutralization, Influenza Virus, *Streptococcus pneumoniae*

## Abstract

An overt pro-inflammatory immune response is a key factor contributing to lethal pneumococcal infection in an influenza pre-infected host and represents a potential target for therapeutic intervention. However, there is a paucity of knowledge about the level of contribution of individual cytokines. Based on the predictions of our previous mathematical modeling approach, the potential benefit of IFN-γ- and/or IL-6-specific antibody-mediated cytokine neutralization was explored in C57BL/6 mice infected with the influenza A/PR/8/34 strain, which were subsequently infected with the *Streptococcus pneumoniae* strain TIGR4 on day 7 post influenza. While single IL-6 neutralization had no effect on respiratory bacterial clearance, single IFN-γ neutralization enhanced local bacterial clearance in the lungs. Concomitant neutralization of IFN-γ and IL-6 significantly reduced the degree of pneumonia as well as bacteremia compared to the control group, indicating a positive effect for the host during secondary bacterial infection. The results of our model-driven experimental study reveal that the predicted therapeutic value of IFN-γ and IL-6 neutralization in secondary pneumococcal infection following influenza infection is tightly dependent on the experimental protocol while at the same time paving the way toward the development of effective immune therapies.

## Introduction

Influenza A virus (IAV) infected individuals are predisposed to severe secondary bacterial infections as observed in a substantial number of fatal cases during influenza outbreaks (1). Such complications due to bacterial super-infection substantially contribute to morbidity and mortality and are frequently caused by the gram-positive bacterium *Streptococcus pneumoniae* (*S. pn*.) (2).

Influenza is a common respiratory pathogen that replicates in alveolar epithelial cells of the respiratory tract. The anti-viral immune response is characterized by high amounts of type I interferons and a network of pro-inflammatory cytokines. Consequentially, viral clearance is accomplished by cytotoxic T cells and antibody responses (3). On the other hand, *S. pn*. is an extracellular bacterial pathogen that can cause a range of diseases, such as pneumonia, otitis media, and meningitis, even though it is also a frequent colonizer of the upper respiratory tract of asymptomatic children and adults. In the lung, clearance of *S. pn*. largely depends on alveolar macrophages (AM), neutrophils, and pro-inflammatory cytokines (4). While for a long time it was believed that breaches in the epithelial barriers caused by influenza infection accounted for enhanced susceptibility to severe pneumococcal infections, today we are aware that this synergism is more complex. Multi-layered immune processes have been identified that disturb efficient anti-bacterial host defense and allow severe bacterial infections to establish in influenza-infected individuals. Dysregulated cytokine responses during the underlying viral infection as well as in response to the secondary bacterial pathogen have been described and include both immune-suppressive as well as exaggerated pro-inflammatory responses (2, 5–7).

Co-infection experiments in mice provided the first evidence that not only the disruption of the alveolar epithelial barrier but also the suppression of AM phagocytic function through interferon-γ (IFN-γ) plays an important role for the fatal synergism between IAV and *S. pn*. (8, 9). It is well-accepted that IFN-γ produced during the viral infection downregulates the expression of the alveolar macrophage receptor with collagenous structure (MARCO) which in turn inhibits AM-mediated microbial clearance and consequently leads to bacterial outgrowth and invasion (8, 10–13). In humans, in addition to inflammatory cytokines, bacterial loads in the co-infected lung were positively associated with the level of the chemokine CXCL-10 (14).

To enable targeted treatment of severe secondary bacterial infections in influenza-infected individuals, a detailed understanding of the underlying disease mechanisms is critical. Mathematical approaches have been a valuable tool in modeling the complex interactions and interdependencies in influenza infection (15–19), pneumococcal infections (20–22), co-infections (23–26), and the respective host immune responses (27–34). Nevertheless, little research has been done in the field of mathematical modeling to better understand inflammatory responses and their targeted neutralization during co-infections with IAV and *S. pn*. (27).

In a data-driven mathematical modeling approach (27), we have addressed the hierarchical contribution of different pro-inflammatory cytokines on bacterial outgrowth in a mouse model of secondary pneumococcal infection following influenza infection and have identified IFN-γ and interleukin-6 (IL-6) dynamics as strong and time-dependent factors for bacterial invasion (27). Our model predicted that neutralization of IFN-γ alone or in combination with IL-6, but not neutralization of IL-6 alone, would restore bacterial clearance in this setting.

The prophylactic and therapeutic strategies currently available in the context of severe secondary bacterial infections in influenza patients are limited and immune-modulation of the dysregulated responses has emerged as a promising approach (35). Therefore, we now explored the predictions of our mathematical modeling approach in a model-driven experimental approach *in vivo*. In order to test *in silico* predictions and exploit the observed therapeutic potential of cytokine-specific neutralizations, antibody-mediated neutralizations of IFN-γ and/or IL-6 were performed *in vivo* post primary influenza infection. In this animal model, C57BL/6 mice were infected with *S. pn*. strain TIGR4 (10^3^ or 10^6^ CFU—colony-forming unit) on day 7 post sub-lethal influenza infection (A/PR/8/34; 0.32 or 0.17 TCID_50_—Median Tissue Culture Infectious Dose). The outcome of these studies provides a rational framework for the development of improved mathematical models and precise as well as effective immune interventions.

## Results

### IFN-γ Neutralization During Secondary Pneumococcal Infection (10^6^ CFU) Following Influenza Infection (0.32 TCID_50_) Leads to a Trend of Reduced Airway Bacterial Burden

**Figure 1:**
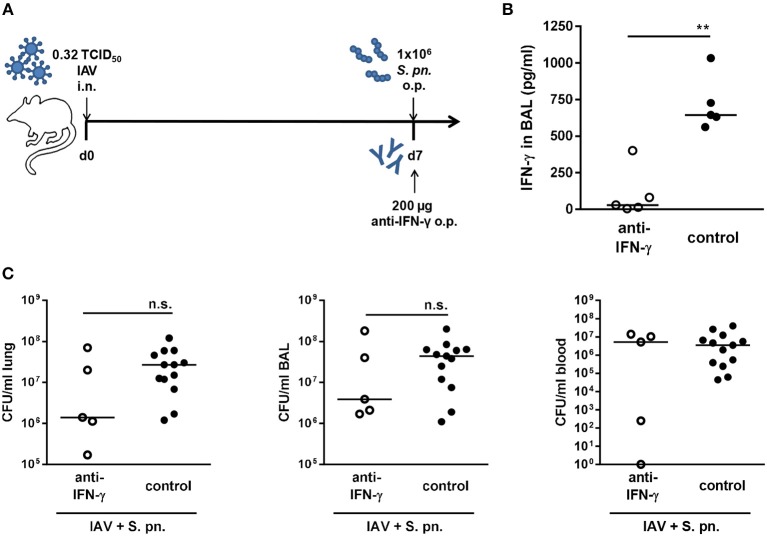
Single IFN-γ neutralization during co-infection with 0.32 TCID_50_ IAV and 1 × 10^6^ CFU *S. pn*. Mice were infected intranasally (i.n.) with 0.32 TCID_50_ IAV on day 0. On day 7, mice were treated with anti-IFN-γ antibody and infected with 1 × 10^6^ CFU *S. pn*. oropharyngeally (o.p.). The control group was infected likewise and treated with an isotype IgG antibody. **(A)** Experimental setup. **(B)** IFN-γ protein levels in bronchoalveolar lavage (BAL) 24 h post antibody treatment and *S. pn*. infection. **(C)** *S. pn*. bacterial counts in lung tissue, BAL and blood 24 h post antibody treatment and *S. pn*. infection. Data are shown for individual mice and lines indicate the median/group. Controls in **(C)** are compiled from independent experiments. Statistical analysis was performed using the unpaired, one-sided Mann-Whitney test [n.s., not significant (*p* > 0.05), ^**^p < 0.01].

For local IFN-γ neutralization, influenza-infected mice were treated with a neutralizing anti-IFN-γ antibody administered to the respiratory tract together with the secondary bacterial infection (Figure 1A), leading to significantly reduced respiratory IFN-γ levels (Figure 1B). At the same time, there was a trend toward a reduced bacterial burden in the lungs and airways of the neutralizing antibody-treated group as compared to the control-treated co-infected animals (Figure 1C). Of note, a high load of bacteria was detected in the blood of only 60% of the anti-IFN-γ antibody treated co-infected mice while 100% of the control-treated co-infected mice showed a comparably high degree of bacteremia (Figure 1C). In conclusion, even though the neutralization of IFN-γ failed to restore bacterial clearance and to significantly reduce bacterial outgrowth in the respiratory tract of co-infected mice as predicted by the mathematical model, we observed a strong trend for a potential therapeutic benefit via reducing the bacterial burden in the airways of co-infected animals.

### IL-6 Neutralization During Secondary Pneumococcal Infection (10^6^ CFU) Following Influenza Infection (0.32 TCID_50_) Leads to Significantly Reduced Bacteremia

**Figure 2:**
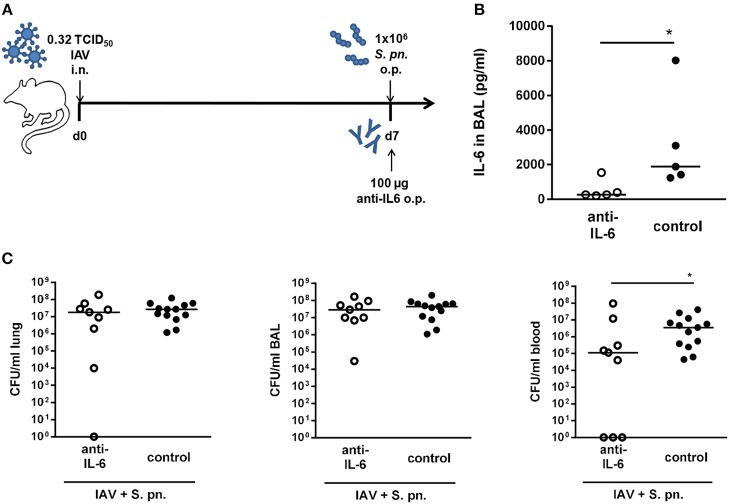
Single IL-6 neutralization during co-infection with 0.32 TCID_50_ IAV and 1 × 10^6^ CFU *S. pn*. Mice were infected intranasally (i.n.) with 0.32 TCID_50_ IAV on day 0. On day 7, mice were treated with anti-IL-6 antibody and infected with 1 × 10^6^ CFU *S. pn*. oropharyngeally (o.p.). The control group was infected likewise and treated with an isotype IgG antibody. **(A)** Experimental setup. **(B)** IL-6 protein levels in bronchoalveolar lavage (BAL) 24 h post antibody treatment and *S. pn*. infection. **(C)** *S. pn*. bacterial counts in lung tissue, BAL and blood 24 h post antibody treatment and *S. pn*. infection. Data are shown for individual mice and lines indicate the median/group. Controls in **(C)** are compiled from independent experiments. Statistical analysis was performed using the unpaired, one-sided Mann-Whitney test (^*^p < 0.05). CFU data are compiled from at least two independent experiments.

Our previous mathematical model (27) predicted a synergistic role for IL-6 in aggravating the detrimental effect of IFN-γ in bacterial outgrowth following secondary pneumococcal infection in IAV infected mice. However, neutralization of IL-6 alone was not predicted to affect bacterial outgrowth or clearance (27). In order to validate these predictions *in vivo*, we also performed antibody-mediated neutralization of IL-6 in co-infected mice. The co-infected mice were treated with a neutralizing IL-6-specific antibody administered to the respiratory tract together with the secondary pneumococcal infection on day 7 post influenza infection (Figure 2A). The anti-IL-6 antibody treatment led to significantly reduced IL-6 levels in the respiratory tract compared to the control-treated co-infected mice (Figure 2B). In contrast to the single IFN-γ neutralization and in line with the predictions of our mathematical model, single neutralization of IL-6 did not have a noticeable effect on the respiratory bacterial burden (Figure 2C). However, surprisingly there was a significant reduction in the systemic bacterial load of the neutralizing antibody-treated mice as compared to the control-treated co-infected mice (Figure 2C). Taken together, while systemic dissemination was significantly less pronounced following the neutralization of IL-6 alone, airway bacterial outgrowth remained unaffected in line with the predictions of the previous mathematical model (27).

### Double Neutralization of IFN-γ and IL-6 During Co-infection Did Not Affect the Bacterial Burden in the Airways Whereas Bacteremia Was Significantly Reduced

**Figure 3:**
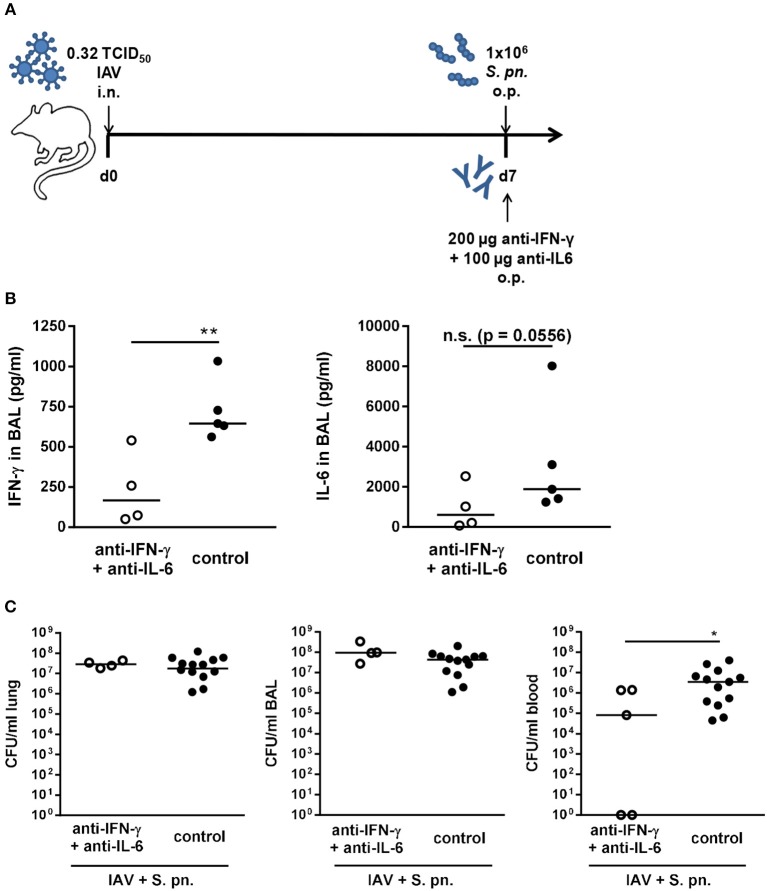
Double-neutralization of IFN-γ and IL-6 during co-infection with 0.32 TCID_50_ IAV and 1 × 10^6^ CFU *S. pn*. Mice were infected intranasally (i.n.) with 0.32 TCID_50_ IAV on day 0. On day 7, mice were treated with anti-IFN-γ antibody together with anti-Il-6 antibody and infected with 1 × 10^6^ CFU *S. pn*. oropharyngeally (o.p.). The control group was infected likewise and treated with an isotype IgG antibody. **(A)** Experimental setup. **(B)** IFN-γ and Il-6 protein levels in bronchoalveolar lavage (BAL) 24 h post antibody treatment and *S. pn*. infection. **(C)** *S. pn*. bacterial counts in lung tissue, BAL and blood 24 h post antibody treatment and *S. pn*. infection. Data are shown for individual mice and lines indicate the median/group. Controls in **(C)** are compiled from independent experiments. Statistical analysis was performed using the unpaired, one-sided Mann-Whitney test [n.s., not significant (*p* > 0.05), ^*^p < 0.05, ^**^p < 0.01]. Data are compiled from at least two independent experiments.

In our previously published mathematical model, the simultaneous neutralization of IFN-γ and IL-6 was highlighted to enhance the positive effect of IFN-γ neutralization alone (27). In order to test this prediction, mice were simultaneously treated with the neutralizing antibodies for both IFN-γ and IL-6 through administration to the respiratory tract during co-infection (Figure 3A). While IFN-γ levels were significantly reduced in the bronchoalveolar lavage (BAL) of the antibody-treated co-infected mice, the reduction of IL-6 levels was substantial despite it did not reach statistical significance (Figure 3B). Nevertheless, as for the neutralization of IL-6 alone, the systemic bacterial burden was significantly reduced following neutralization of both IFN-γ and IL-6 as compared to the control-treated co-infected mice (Figure 3C). However, the administration of anti-IL-6 antibody together with the anti-IFN-γ antibody did not reduce the bacterial burden in the respiratory tract of co-infected mice (Figure 3C) and thereby did not recapitulate the predictions of the mathematical model.

### Single or Simultaneous Neutralization of IFN-γ and IL-6 Did Not Have Beneficial Effects in a Co-infection Model Employing a Reduced Pneumococcal Dose (10^3^ CFU)

**Figure 4:**
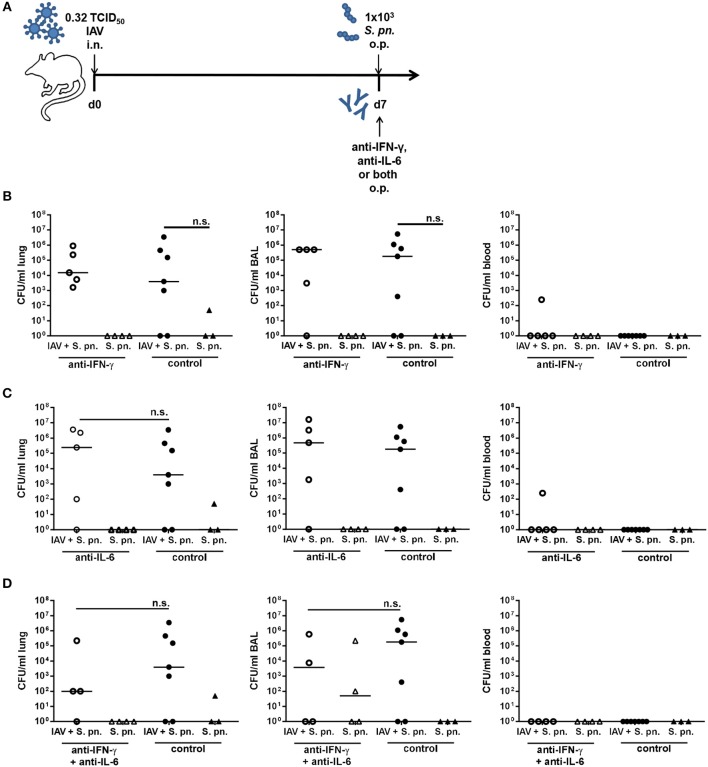
Neutralization of IFN-γ and/or IL-6 during co-infection with 0.32 TCID_50_ IAV and 1 × 10^3^ CFU *S. pn*. Mice were infected intranasally (i.n.) with 0.32 TCID_50_ IAV or left uninfected on day 0. On day 7, mice were treated with anti-IFN-γ antibody **(B)**, anti-IL-6 antibody **(C)**, or both **(D)** and infected with 1 × 10^3^ CFU *S. pn*. oropharyngeally (o.p.). The control groups were infected likewise and treated with an isotype IgG antibody. **(A)** Experimental setup. **(B–D)** *S. pn*. bacterial counts in lung tissue, BAL and blood 24 h post antibody treatment and *S. pn*. infection. Data are shown for individual mice and lines indicate the median/group. Co-infected, antibody-treated and co-infected control groups were compared using the unpaired, one-sided Mann-Whitney test [n.s., not significant (*p* > 0.05)].

In the utilized co-infection model pneumococcal outgrowth in the respiratory tract is severe and reaches extremely high bacterial counts as early as 18 h post the secondary infection. Therefore, neutralization of IFN-γ and/or IL-6 was performed in a low bacterial dose co-infection model (1 × 10^3^ CFU). To this end, mice were infected with 0.32 TCID_50_ IAV as described above, co-infected with 1 × 10^3^ CFU *S. pn.*, and at the same time treated through the administration of unchanged doses of neutralizing antibodies specific for IFN-γ and/or IL-6 to the respiratory tract (Figure 4A). As expected, in this co-infection model, the lower dose of *S. pn*. was associated with a lower grade of bacterial pneumonia (1 × 10^3^-10^6^ CFU) in contrast to the high-grade pneumonia (1 × 10^7^-10^8^ CFU) observed post co-infection with 1 × 10^6^ CFU *S. pn*. (Figure 4B). Of note, there was a clear and strong increase in the respiratory bacterial burden in 1 × 10^3^ CFU *S. pn*. co-infected as compared to only *S. pn*. infected mice, which was however not statistically significant. Also, no consistent dissemination of bacteria to the circulation was detectable in the co-infected mice. In this model, the administration of the neutralizing antibodies specific for IFN-γ or IL-6 alone did not have any beneficial effects on bacterial clearance in either case, as the respiratory bacterial burden was unchanged between neutralizing antibody-treated and control-treated co-infected mice (Figures 4B,C). Importantly, however, a trend for a reduced bacterial burden was observed in the airways post simultaneous neutralization of IFN-γ and IL-6 in the co-infected mice as compared to control-treated co-infected animals (Figure 4D). Nevertheless, taken together antibody-mediated neutralization of IL-6 or IFN-γ as well as simultaneous neutralization of both in a co-infection model utilizing a reduced bacterial infectious dose also did not show the clear effects on bacterial outgrowth and clearance predicted by our previously mathematical model.

### Repeated Neutralization of IFN-γ Alone or in Combination With IL-6 Significantly Reduces the Lung Bacterial Burden in Secondary Pneumococcal Infection in a Co-infection Model Based on Low Infectious-Dose IAV Infection (0.17 TCID_50_)

**Figure 5:**
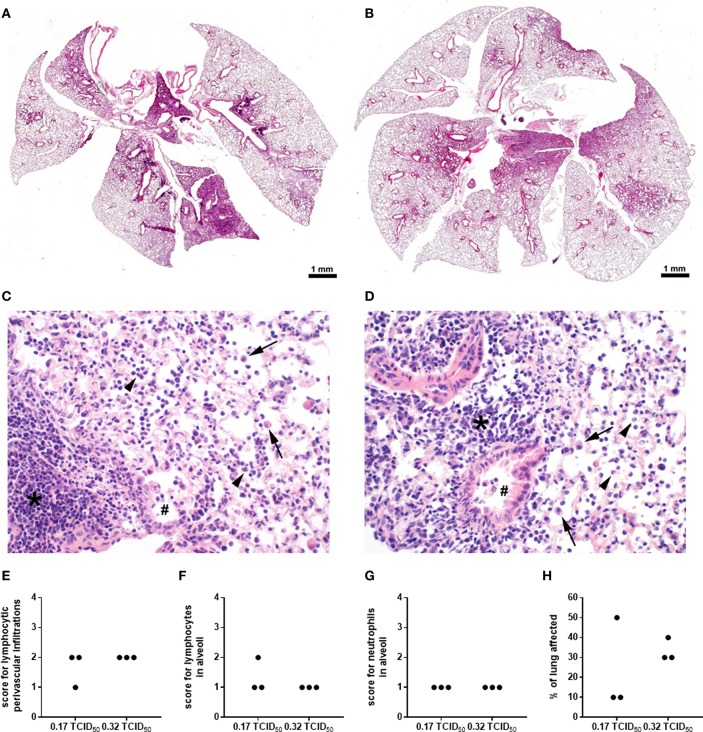
Histopathological characterization of high and low dose IAV infection. WT C57BL/6J mice were intranasally infected with the A/PR8/34 H1N1 strain of IAV. Images show a representative overview **(A,B)** and details **(C,D)**; 400x) of the histopathological changes observed in the lungs on day 7 post infection with 0.32 **(A,C)** or 0.17 **(B,D)** TCID_50_ IAV following H&E staining. Bronchi (double crosses) contain few activated macrophages and the bronchiolar epithelium is attenuated and partially sloughed into the lumen. Alveoli are filled by sloughed epithelial cells, macrophages (arrows), degenerated neutrophils (arrow heads), and cellular debris. The alveolar septae and the perivascular and peribronchial interstitium are expanded by neutrophils, macrophages and lymphocytes (asterisks). Lymphocytic perivascular infiltrations **(E)** and the number of lymphocytes **(F)** and neutrophils **(G)** in the alveoli were scored on a scale from 1 to 4 and the percent of the lung affected by the infection **(H)** was evaluated and are shown for individual animals.

In order to further characterize the effects of IFN-γ and/or IL-6 neutralization on secondary pneumococcal infection following IAV infection, the established co-infection model was furthermore adjusted by reducing the infectious dose in the underlying IAV infection. In order to compare the pathological features of the two different IAV infectious doses employed in our study, pulmonary histopathology was performed on day 7 post infection with 0.32 and 0.17 TCID_50_ IAV (Figure 5). Overview images show a partial consolidation of the lung, predominantly close to the hilus and affecting the parenchyma surrounding the large bronchi (Figures 5A,B). In detail, major histological hallmarks for IAV infection were observed for both infectious doses, which were the accumulation of sloughed epithelial cells, macrophages and degenerated neutrophils in the alveoli and bronchiolar lumina as well as lymphocyte, macrophage and neutrophil infiltration in the alveolar septae and the perivascular as well as peribronchial interstitium (Figures 5C,D).

**Figure 6:**
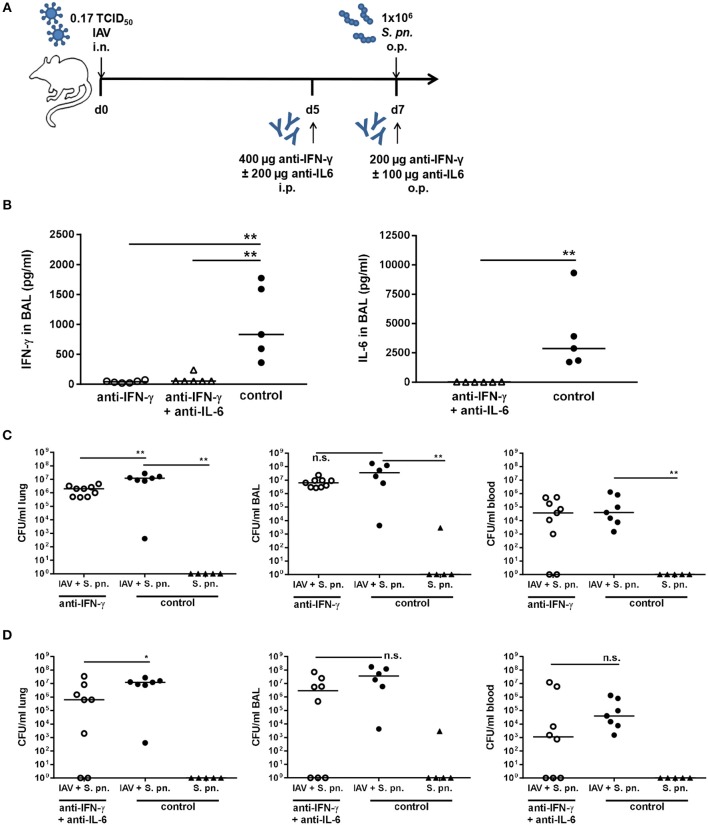
Neutralization of IFN-γ and double-neutralization of IFN-γ and IL-6 during co-infection with 0.17 TCID_50_ IAV and 1 × 10^6^ CFU *S. pn*. significantly reduced the lung bacterial burden. Mice were infected intranasally (i.n.) with 0.17 TCID_50_ IAV or left uninfected on day 0. On day 5, mice were intraperitoneally (i.p.) injected with anti-IFN-γ antibody alone or in combination with anti-IL-6 antibody. On day 7, mice were treated with anti-IFN-γ antibody alone or in combination with anti-IL-6 antibody and at the same time infected with 1 × 10^6^ CFU *S. p.n*. oropharyngeally (o.p.). The control groups were infected likewise and treated with an isotype IgG antibody. **(A)** Experimental setup. **(B)** IFN-γ and IL-6 protein levels in bronchoalveolar lavage (BAL) 24 h post antibody treatment and *S. pn*. infection. **(C,D)** *S. pn*. bacterial counts in lung tissue, BAL and blood 24 h post antibody treatment and *S. pn*. infection. Data are shown for individual mice and lines indicate the median/group. Co-infected, neutralizing antibody-treated were compared to co-infected, control-antibody-treated groups, and co-infected, control-antibody-treated groups were compared to single-infected, control-antibody-treated groups using the unpaired, one-sided Mann-Whitney test [n.s. = not significant (*p* > 0.05), ^*^p < 0.05, ^**^p < 0.01]. Data from co-infected mice are compiled from at least two independent experiments.

The inflammatory lesions were distributed multi-focally or focally extensively and corresponded to the pattern of a sub-acute broncho-interstitial pneumonia. Scorings of the histopathological changes revealed little alteration between the two viral doses (Figures 5E–G), whereas the percent of lung tissue affected was on average smaller following infection with 0.17 as compared to 0.32 TCID_50_ IAV (Figure 5H). At the same time, the lungs of PBS-treated mice did not show any pathological findings (data not shown). A reduction of the overall severity of the underlying IAV infection monitored as relative body weight loss (data not shown) also enabled the repeated administration of the neutralizing antibodies. Therefore, mice were infected with 0.17 TCID_50_ IAV on day 0, neutralizing antibodies specific for either IFN-γ alone or IFN-γ and IL-6 were intraperitoneally injected on day 5, followed by secondary pneumococcal infection with 1 × 10^6^ CFU of *S. pn*. and the simultaneous administration of the respective neutralizing antibodies to the respiratory tract on day 7 (Figure 6A). In this model, neutralizations were only performed for IFN-γ alone or in combination with IL-6 as these scenarios were predicted to reduce bacterial outgrowth in the respiratory tract of co-infected mice in the underlying mathematical model (27). At the same time, the previous experiments confirmed that neutralization of IL-6 alone did not have a positive effect on the respiratory bacterial burden in secondary pneumococcal infection following influenza infection. While lowering the viral dose did not lead to a substantial reduction in the airway levels of IFN-γ and IL-6 detected 24 h following co-infection, neutralization was exceptionally efficient following the administration of the respective antibodies intraperitoneally on day 5 and to the respiratory tract on day 7 post influenza infection (Figure 6B). Of note, secondary pneumococcal infection with 1 × 10^6^ CFU *S. pn*. following infection with 0.17 TCID_50_ IAV led to a significant bacterial outgrowth in the respiratory tract as well as to significantly increased systemic dissemination as compared to pneumococcal infection alone (Figure 6C). Importantly, in this co-infection model, the neutralization of IFN-γ alone as well as the simultaneous neutralization of IFN-γ and IL-6 led to a significant reduction of the lung bacterial load in co-infected mice as compared to the control-treated co-infected mice (Figures 6C,D). Although there was no complete bacterial clearance, these observations clearly confirm the predictions of the mathematical model regarding the beneficial potential of neutralizing IFN-γ alone or in combination with IL-6 for the restoration of disrupted anti-bacterial defense in the respiratory tract during secondary pneumococcal infection following influenza infection.

### Neutralization of IFN-γ and IL-6 Alters the Levels of Additional Cytokines in the Respiratory Tract

**Figure 7:**
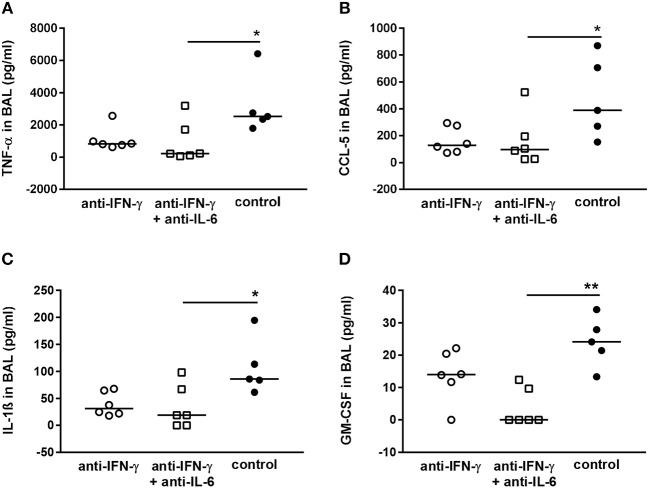
Double-neutralization of IFN-γ and IL-6 during co-infection with 0.17 TCID_50_ IAV and 1 × 10^6^ CFU *S. pn*. significantly affects TNF-α, CCL-5, Il-1ß, and GM-CSF protein levels in BAL. Mice were infected intranasally (i.n.) with 0.17 TCID_50_ IAV on day 0. On day 5, mice were intraperitoneally (i.p.) injected with anti-IFN-γ antibody alone or in combination with anti-Il-6 antibody. On day 7, mice were treated with anti-IFN-γ antibody alone or in combination with anti-IL-6 antibody oropharyngeally (o.p.) and at the same time infected with 1 × 10^6^ CFU *S. pn*. o.p. The control group was treated with an isotype IgG antibody and infected likewise. **(A–D)** Protein levels of TNF-α, CCL-5, Il-1ß, and GM-CSF in bronchoalveolar lavage (BAL) 24 h post o.p. antibody treatment and *S. pn*. infection. Data are shown for individual animals indicating the median/group and are compiled from two independent experiments. Neutralizing antibody-treated groups were compared to the control antibody-treated group using the unpaired Kruskal-Wallis test with Dunn's test for multiple comparisons (^*^p < 0.05, ^**^p < 0.01).

Although in principle we confirmed the beneficial effect of neutralizing IFN-γ and IL-6 for the host regarding the respiratory bacterial burden in secondary pneumococcal infection following influenza infection, there were substantial discrepancies between the predictions of mathematical modeling and our experimental results when attempting to validate these predictions *in vivo*. One possible reason is the secondary effects of the neutralization of single cytokines on the immunological network that have not been considered in our modeling approach. In order to evaluate the effect of neutralization of the targeted cytokines IFN-γ and IL-6 on the network of pro-inflammatory mediators produced in the airways in response to co-infection, the levels of TNF-α, CCL-5, IL-1ß, GM-CSF, IL-10, IFN-α, IFN-ß, CXCL-10, IL-12p70, CCL-2, and CXCL-1 were determined. Neutralization of IFN-γ and/or IL-6 during co-infection with 0.31 TCID_50_ IAV and 10^6^ CFU *S. pn*. did not significantly change the airway levels of these mediators (Supplementary Figure 1). However, in the co-infection mouse model employing the reduced viral dose, the neutralization of IFN-γ and IFN-γ together with IL-6 had clear effects on the concentrations of TNF-α (Figure 7A), CCL-5 (Figure 7B), IL-1ß (Figure 7C), and GM-CSF (Figure 7D). A substantial reduction in the levels of these cytokines was observed following single neutralization of IFN-γ, whereas the simultaneous neutralization of IFN-γ and IL-6 in co-infected mice led to significantly reduced levels. In contrast, IL-10, IFN-α, IFN-ß, CXCL-10, IL-12p70, CCL-2, and CXCL-1 levels were also not significantly affected by IFN-γ and IFN-γ/IL-6 neutralization in co-infection with 0.17 TCID_50_ IAV and 1 × 10^6^ CFU *S. pn*. (Supplementary Figure 2). These observations clearly show that next to its potential to positively affect bacterial clearance the neutralization of specific cytokines during co-infection can have multi-layered immune regulatory effects that also need to be reflected by mathematical predictions to make them more precise.

## Discussion

In our study, we tested predictions from an experimental data-driven mathematical modeling approach *in vivo*. To this end we assessed the effect of the neutralization of IFN-γ and/or IL-6 during co-infection with IAV and *S. pn*. on the bacterial load in the respiratory tract and the blood. Importantly, in this modeling-driven experimental study, we initially employed the established co-infection model that had been used before to generate the data underlying the mathematical model. Here, levels of IFN-γ and IL-6 peaked on day 7 following IAV infection (Supplementary Figure 3) and therefore this time-point has been chosen to evaluate the effects of their neutralization.

However, single respiratory tract IFN-γ neutralization on day 7 post IAV infection, performed concomitant with the bacterial infection, largely failed to recapitulate the mathematical model predictions (27). Interestingly, however, treatment with the IL-6 neutralizing antibody alone as well as in combination with the IFN-γ neutralizing antibody led to significantly reduced systemic bacterial burdens in co-infected mice. Strikingly, simultaneous neutralization of IFN-γ and IL-6 was less efficient with respect to reducing IL-6 levels than neutralization of IL-6 alone. Most likely, this observation was due to secondary effects of the additional neutralization of IFN-γ on IL-6 production or pathology, which in turn lead to altered inflammatory conditions and responses, Ultimately, future studies will be needed to clarify a possible distinct role for IL-6 in secondary pneumococcal infection following IAV infection and here especially in bacterial dissemination from the respiratory tract.

Based on our findings in the original co-infection setting, two time-points of neutralization, i.e., one intraperitoneal injection on day 5 and one administration via the respiratory route on day 7 post IAV infection, were tested in an adjusted infection model. Even though neutralization of IFN-γ has been reported to have only minimal effects on the course of IAV infection (8), in our co-infection models using 0.32 TCID_50_ IAV it adversely affected the general condition of the animals as reflected by overt body weight loss (data not shown). Therefore, the repetitive neutralizations were performed in a co-infection model utilizing a reduced viral load. Primary infection with a reduced viral dose of 0.17 TCID_50_ IAV led to similar histopathological changes and, importantly, still significantly increased susceptibility to secondary pneumococcal infection including systemic dissemination on day 7 post influenza infection.

Consistent with previous co-infection experiments by Smith et al. (23) our results therefore show that the initial IAV infectious dose does not strongly affect the secondary bacterial outgrowth and invasion. In line with this, respiratory IFN-γ and IL-6 levels were similarly high or even higher in pneumococcal co-infection following infection with 0.17 TCID_50_ IAV. Importantly, in this experimental system of secondary pneumococcal infection following infection with a reduced dose of IAV as well as repeated cytokine neutralization, the degree of bacteremia, as well as the grade of pneumonia, was significantly reduced as compared to the respective control group, indicating a positive effect on the response to secondary bacterial infection. Future studies will have to unravel in detail whether the lower viral dose, the higher doses of the neutralizing antibodies, their repeated administration via the intraperitoneal as well as the respiratory route or the effects on other cytokines are key to the significant beneficial effects observed. On the one hand, while both viral doses significantly predisposed for secondary pneumococcal outgrowth and dissemination, the extent of lung tissue affected by the viral infection itself was dose-dependent, which consequently may have influenced the efficacy of neutralizations scenarios. On the other hand, together with reducing the viral dose also the doses of the neutralizing antibodies were increased and led to a more efficient neutralization of IL-6 in the combinatorial neutralization of IFN-γ and IL-6. At the same time, these higher neutralizing antibody doses were administered on days 5 and 7 post IAV infection as compared to a single administration in co-infections following 0.32 TCID_50_ IAV. Of note, both intraperitoneal, as well as respiratory tract-directed administration, have been described for the administration of antibody-treatments in pulmonary studies (8, 36). For multiple administrations of the higher antibody doses, the intraperitoneal injection was chosen to avoid additional anesthesia. Taken together, our results in principle confirm the predictions of our mathematical model, i.e., the potential of neutralizing IFN-γ alone and in combination with IL-6 for restoring anti-bacterial host defense in secondary pneumococcal infection following IAV infection. At the same time, the clear synergistic effect for simultaneously neutralizing IFN-γ and IL-6, as predicted by the mathematical model, was not observed *in vivo* as additional neutralization of IL-6 abolished the positive trend observed for IFN-γ neutralization following co-infection with 0.32 TCID_50_ IAV and 1 × 10^6^ CFU *S. pn*. Ultimately, the detailed inter-dependencies will have to be addressed together with the question, how the observed benefits can be extended and whether they can be translated to decreased morbidity and mortality in secondary bacterial infection, possibly also in combination with antibiotic treatment (37).

Counterintuitively, a reduction in the bacterial dose had little influence on the effect of the cytokine neutralization. By a factor of 1000 reduction in bacterial dose was tested to evaluate if the positive effect of double neutralization predicted by the mathematical model could be achieved. However, the bacterial load remained unchanged between the neutralizing antibody-treated and the control-treated co-infected groups (Figure 4). While the neutralization of IFN-γ and IL-6 alone led to marginally increased bacterial loads in the respiratory tract of co-infected mice, their simultaneous neutralization led to a small trend for decreased respiratory bacterial burdens. Ultimately, there was however not even a significant increase in the bacterial burden following secondary pneumococcal infection at a dose of 1 × 10^3^ CFU on day 7 post IAV infection with 0.32 TCID_50_, suggesting a threshold bacterial dose is necessary to establish severe secondary infection including systemic dissemination. Of note, this significant synergism was clearly observed in co-infection with 1 × 10^6^ CFU *S.pn*. on day 7 post infection with 0.17 TCID_50_ IAV.

Our original mathematical model (27) predicted the restoration of bacterial clearance through the neutralization of IFN-γ alone and in combination with IL-6 during co-infection with 0.32 TCID_50_ IAV and 1 × 10^6^ CFU *S. pn*. While we observed trends as well as significant decreases in respiratory bacterial loads and systemic dissemination in the different experimental approaches of our study, there is no consistent evidence of restored bacterial clearance. These results were therefore behind the predictions and expectations from the mathematical modeling and the *in silico* cytokine neutralizations. In particular, the mathematical models (27) did not consider that neutralization protocols would have a delay to promote innate immune cells to recover their phagocytosis efficiency. Furthermore, we show that simultaneous neutralization of IFN-γ and IL-6 can have clear and significant effects on levels of other pro-inflammatory mediators, thereby profoundly altering the local inflammatory milieu and immune network, possibly also affecting viral clearance. As an example, neutralization of IFN-γ and IL-6 during co-infection with 0.17 TCID_50_ IAV and 1 × 10^6^ CFU *S. pn*. significantly decreased GM-CSF levels. Its overexpression in turn has been shown to prevent mortality and modulate macrophage polarization in IAV infection in mice (38) and inhaled GM-CSF therapy has been shown to have benefits in patients with pneumonia-associated acute respiratory distress syndrome (39). Such effects were however not part of the underlying mathematical model, whereas they most likely do have direct or indirect effects on the mechanisms of anti-bacterial defense in co-infection. Furthermore, these findings demonstrate potentially harmful effects for neutralizing single or multiple cytokines that need to be taken into account. While mice deficient in IFN-γ signaling have been shown to effectively respond to IAV infection (8), a crucial role for IL-6 has been described (40, 41). Of note, multiple time-point neutralization of IFN-γ following IAV infection with 0.32 TCID_50_ increased morbidity in our system (data not shown). Therefore, exact knowledge of the balance between beneficial and detrimental cytokine effects during IAV and co-infection will be essential for further evaluation of the therapeutic potential of neutralizations and likewise for the development of precise models. Of note, while in co-infections the detrimental effects of IFN-γ have been described (8), this is less clear for IL-6 but suggested by the positive effect of IL-6 neutralization on systemic dissemination.

**Figure 8:**
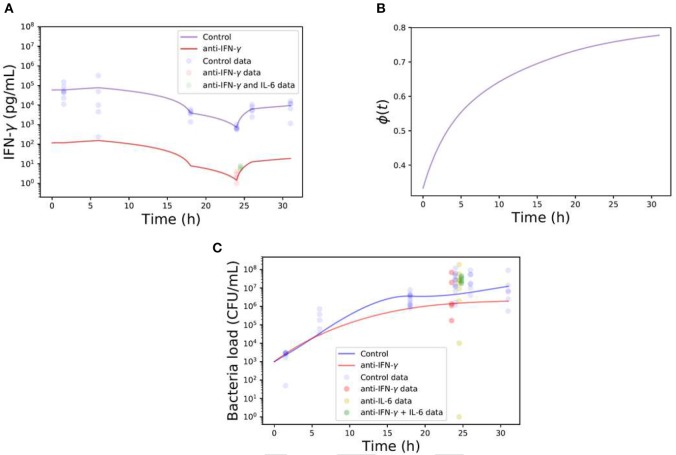
Computational simulations of single or double-neutralization of IFN-γ and IL-6 during co-infection with 0.32 TCID_50_ IAV and 1 × 10^6^ CFU *S. pn*. **(A)** The blue line represents the IFN-γ kinetics during co-infection (control) and the red line shows the IFN-γ concentration kinetics based on the neutralization protocol (~500-fold reduction respect co-infection). Note that the continuous blue line is built with a piecewise linear function of the experimental data points. The red line is based on the control case but adjusted to pass by the time point of anti-IFN- γ experiments. **(B)** Bacterial clearance function ϕ (Equation 5) during neutralization protocol of IFN-γ. **(C)** The blue line represents the bacterial load in the lungs during co-infection (control) while the red line displays the neutralization protocol. The different color dots represent the data of different neutralization protocols.

Our findings have shed light into the multitude of inter-related immune-regulatory pathways in action in a co-infection setting, drawing a more complex picture for mathematical modeling. Even though the levels of IFN-γ following neutralization (~500-fold reduction reached 24 h post antibody treatment) were consistent during the time of co-infection (Figure 8A), refined mathematical simulations in Figure 8B show a strong time dependency to recover necessary levels of phagocytosis to avoid a bacterial invasion as depicted in Figure 8C. However, with the data available from our different approaches for cytokine neutralization and co-infection, these modeling results can only be taken in a qualitative form.

Note that also our neutralization protocols may have different limitations to differences in mouse strains. A very recent study by Metzger's group (12) presented data that BALB/c mice exhibited a strong IFN-γ dependent reduction in AM numbers post influenza infection. On the other hand, AM levels in C57BL/6 mice were maintained throughout the course of influenza infection, however, cells displayed an altered phenotype. These mouse strain-dependent differences were observed regardless of viral strains or whether the infection was performed with low or high doses. However, the mechanisms responsible for differential AM responses in BALB/c and C57BL/6 mice are unknown (12). The most conceivable theory is that immune responses in C57BL/6 mice are preferentially skewed toward the Th1 and M1 lineage, whereas BALB/c mice are believed to be prone to develop Th2- and M2-dominated responses (42, 43). Ultimately, AM depletion was rescued in IFN-γ^−/−^ BALB/c mice improving bacterial clearance and disease outcome in this mouse strain (8, 12). Thus, these findings together with our study underline the multitude of factors, including skews in the host immune response, to be considered for designing reliable mathematical models and for designing effective and precise therapeutic approaches.

Even though the previous *in silico* predictions of our mathematical model were not fully met *in vivo*, the results of this model-driven experimental study emphasize the potential and at the same time illustrate the limitations of modeling approaches, thereby significantly contributing to the current knowledge and building a basis for future approaches translating improved mathematical models into potential therapies.

## Methods

### Mice

Nine to eleven weeks old C57BL/6JOlaHsd female mice were purchased from Envigo (formerly known as Harlan Laboratories, Venray, Netherlands) and were housed in a specific pathogen-free environment according to the guidelines of the regional animal care committee. All the experiments were approved and conducted in accordance to the guidelines set by the local animal ethical bodies for the Helmholtz Centre for Infection Research (Niedersächsisches Landesamt für Verbraucherschutz und Lebensmittelsicherheit, animal permit code: 16/2319). Mice were age-matched for all the experiments. All mice were closely monitored and scored for weight loss, posture, pilo-erection, respiration, eye discharge, redness of the eye conjunctiva and response to stimulus. Animals with severe symptoms were euthanized and the infection was considered as lethal.

### Bacterial and Viral Preparations

The pneumococcal serotype 4 strain TIGR4 (T4; ATCC BAA-334) was used. Bacteria were grown to the mid-logarithmic growth phase (optical density of 0.35 at 620 nm) in freshly prepared pre-warmed THY medium (THB Sigma-Aldrich, Germany and yeast extract, Roth, Germany) at 37°C in a water bath and were harvested by adding 10% v/v glycerol (Roth, Germany) before storage at −70°C. For mouse infections, the frozen stocks were thawed, centrifuged, washed once in 1 ml of phosphate-buffered saline (PBS, Gibco, UK) and subsequently pelleted before diluting to the desired concentration. The challenge dose was confirmed for each infection by plating 10-fold serial dilutions on blood agar plates (BD Diagnostic Systems, Columbia Agar with 5% sheep blood, Germany) and overnight incubation at 37°C and 5% CO_2_.

For IAV infection, the viral strain PR8/A/34 had been previously produced in adherent Madin-Darby canine kidney (MDCK) cells (44). The 50% tissue culture infectious dose (TCID_50_) was determined by incubating 10-fold serial dilutions of the viral stock on MDCK cells in Dulbecco's modified eagle medium (DMEM; Life Technologies, Germany) supplemented with 0.0002% trypsin (Sigma-Aldrich, Germany) and 1% penicillin/streptomycin (ThermoFisher, USA) for 5 days followed by addition of 0.5% chicken red blood cells (Fiebig Naehrstofftechnik, Germany) to the culture medium. Agglutination of the red blood cells was documented and the TCID_50_ was calculated using the endpoint calculation by Reed and Muench (45).

### Mouse Infection Models

Prior to infection, all mice were weighed and anesthetized through intraperitoneal administration of a ketamine (WdT, Germany; 10 g/100 ml) and xylazine solution (Bayer, Germany; 2 g/100 ml) at a dose of 0.1 ml/10 g mouse body weight.

*S. pn*. was administered to the respiratory tract through oropharyngeal instillation (46). Mice were placed on their back on an intubation slope, the mouth was held open and the tongue gently placed to the side of the mouth using flat head forceps. A cold light lamp with a flexible light guide was used to illuminate the anterior pharynx and the challenge dose of 1 × 10^6^ or 1 × 10^3^ colony forming units (CFU) *S. pn*. in 25 μl PBS was instilled into the laryngopharynx using a flexible gel loading pipet tip (Corning Inc., USA). After application of the inoculum, the nares were manually blocked for 5 s to ensure breathing through the mouth and aspiration of the inoculum into the trachea. Control groups received 25 μl of sterile PBS. For the intranasal instillation of IAV, mice were held upright with the head slightly tilted back and the inoculum was administered dropwise to the nares in a volume of 25 μl sterile PBS.

### Antibody-Mediated Cytokine Neutralization Protocols

Antibody-mediated neutralization of IFN-γ and IL-6 was performed using the rat anti-mouse IFN-γ antibody clone AN-18 (FPLC-purified from hybridoma) and the rat anti-mouse IL-6 antibody clone MP5-20F3 (Bio X Cell), respectively. A rat control IgG1 isotype (TNP6A7; Bio X Cell) was used as control antibody. All antibodies were sterile, azide-free and low-endotoxin, suitable for *in vivo* administration. Mice infected with 0.32 TCID_50_ IAV were treated with 200 μg anti-IFN-γ and/or 100 μg anti-IL-6 antibody oropharyngeally on day 7 post-IAV infection together with the secondary bacterial infection to deliver the neutralizing antibodies to the respiratory tract. Mice infected with 0.17 TCID_50_ IAV were treated intraperitoneally with 400 μg anti-IFN-γ antibody alone or in combination with 200 μg anti-IL-6 antibody on day 5 post influenza infection. On day 7 post influenza infection, these mice were oropharyngeally treated with 200 μg anti-IFN-γ antibody alone or in combination with 100 μg anti-IL-6 antibody together with the secondary bacterial infection.

### Assessment of the Bacterial Burden

Animals were sacrificed using CO_2_ inhalation. BAL was obtained by flushing the lungs once with 1 ml PBS via the trachea using a 22G indwelling cannula (Braun, Germany). Following perfusion, the lungs were excised, and the tissue was mechanically homogenized in 1 ml PBS by passing through a 70 μm filter (Corning Inc., USA). Cardiac blood was collected and diluted in PBS. Bacterial CFU were determined by plating serial dilutions of BAL, post-lavage lung homogenates and blood on blood agar plates. The plates were incubated for 16–18 h at 37°C and 5% CO_2_, CFU were manually counted and the CFU per ml were calculated.

### Detection of Cytokines in BAL

An enzyme-linked immunosorbent assay (ELISA) was used to determine the protein concentrations of IL-6. A 96-well Nunc ELISA plate (F96 Maxisorp Nunc-immuno plate, Thermo Scientific) was coated with 0.5 μg/ml of the capture antibody MP5-20F3 (Bio X Cell). Washing was performed with the Power Washer (Tecan). To prevent unspecific binding, 200 μl of blocking buffer were added and the plate was incubated on a shaker for 1 h at room temperature. Subsequently, 50 μl of sample were added and incubated for 2 h at room temperature on a shaker. An IL-6 protein standard (recombinant mouse IL-6, BioLegend) was used at concentrations of 500, 250, 125, 61.5, 32.3, 15.6, and 7.6 pg/ml. After the incubation, the plate was washed and incubated with 50 μl of the detection antibody (1.25 μg/ml; MP532C11, BD Pharmingen) for 1 h. This incubation was followed by washing and adding 50 μl of 1:2000 diluted Streptavidin-horse radish peroxidase (BD Pharmingen). The plate was incubated for 30–45 min in the dark and washed before adding 50 μl of TMB substrate (BIOZOL). Following the addition of 50 μl ELISA stop solution (15 min) the absorption at 450 nm was read by using a photometer (TECAN Sunrise). The background absorption was read at 570 nm as the reference.

For the detection of IFN-γ protein levels through ELISA, a similar protocol was followed. The capture antibody (AN-18; FPLC-purified from hybridoma) was used at a concentration of 2.7 μg/ml, the standard was the recombinant mouse IFN-γ (BioLegend) and the detection antibody (R4-6A2; FPLC-purified from hybridoma) was diluted to a working concentration of 0.6 μg/ml. For the simultaneous detection of IL-6, IFN-γ, TNF-α, CCL-5, IL-1ß, GM-CSF, IL-10, IFN-α, IFN-ß, CXCL-10, IL-12p70, CCL-2, and CXCL-1 in BAL samples a flow-cytometry based multiplex detection assay (LegendPlex mouse anti-virus response panel, BioLegend) was used according to the manufacturer's recommendations.

### Histopathological Analysis

Lungs were fixed in 4% formalin and routinely embedded in paraffin. Sections were cut 5 μm thick, dewaxed, and stained with hematoxylin and eosin (H&E). A blinded histopathological evaluation was performed by a veterinary pathologist certified by the European College of Veterinary Pathologists.

### Mathematical Modeling

To understand the limitation of previous predictions (27), we employ as a starting point our previous mathematical work (27) that fitted best to the co-infection experimental data and that is

(1) $$\begin{array}{r} \frac{dB\left(t\right)}{dt}=r\left(1-\frac{B\left(t\right)}{K_{B}}\right)B\left(t\right)-\;c_{B}f_{x}\left(t\right)B\left(t\right), \end{array}$$

where *r* (1.13 h^−1^) is the bacterial proliferation rate with a maximum carrying capacity K_B_ (2.3 × 10^8^ CFU/ml). Phagocytosis of the bacteria is considered by the multiplicative term *c*_*b*_*f*_*x*_, where c_b_ (1.28 h^−1^) is the constant phagocytosis rate. The term *f*_*x*_ is the mathematical function which served to test different hypotheses. The biological meaning of *f*_*x*_is the bacterial clearance inhibition provided by the inflammation to alveolar macrophages (AMs). In this work, we use here the model M7 from previous mathematical work (27), which was one of the best functions that fitted co-infected data, that is

(2) $$\begin{array}{r} f_{x}\left(t\right)=\left(\frac{A_{1}}{IL-6\left(t\right)+A_{1}}\right)\left(\frac{A_{2}}{IFN-\gamma \left(t\right)+A_{2}}\right), \end{array}$$

where A_1_ = 4.05 x 10^7^ and A_2_ = 5.46 x 10^4^. Note that the terms for IL-6(t) and IFN-γ(t) are not modeled mechanistically but as piecewise linear functions from the experimental data (27), which is considered as an input to the Equation (2).

Our previous modeling work assumed that pro-inflammatory cytokines directly affect bacterial clearance. However, there may be a time interval where immune cells responsible (e.g., macrophages) for bacterial clearance return to levels that sufficiently clear the bacterial infection. Therefore, the importance to model kinetically the dynamics of macrophages. Thus, the mathematical model is extended here to

(3) $$\begin{array}{rcl} \frac{dB\left(t\right)}{dt} & = & r\left(1-\frac{B\left(t\right)}{K_{B}}\right)B\left(t\right)-\;c_{B}\phi \left(t\right)B\left(t\right), \end{array}$$

(4) $$\begin{array}{rcl} \frac{dM\left(t\right)}{dt} & = & s_{M}-d_{M}M\left(t\right)-\;f_{x}\left(t\right)M\left(t\right), \end{array}$$

(5) $$\begin{array}{rcl} \phi \left(t\right) & = & \left(\frac{M\left(t\right)}{M\left(t\right)+\theta }\right), \end{array}$$

where M(t) represents the alveolar macrophages (AM) cells that are mainly responsible for bacterial clearance (47). The rationale of Equation (4) is based on previous modeling works (20) to represent the dynamics of alveolar macrophages that is considered as the main component of the innate immune system to encounter the bacterial infection when entering the airway. It is assumed a constant influx of macrophages with the term *S*_*M*_ and a death rate *d*_*M*_. Parameter values are *s*_*M*_ = *M*(0)*d*_*M*_, and *d*_*M*_ = 1/25 h^−1^ as reported by Smith et al. (20).

While in the naive conditions (free infection) it is assumed not any changes of AM, that is M(0) = 1, the number of active macrophages is decreased during influenza infection as reported in previous experimental studies (10). In a similar direction to previous modeling (24), we consider a simple monotone increasing function with respect to AM dynamics. Note that other more complex terms could have been used for ∅(*t*), however, due to the limitations in the number of data points during neutralizations we consider the simple monotone term. Smith et al. (24) derived a critical non-linear threshold which dictates the bacterial invasion during co-infection, which is about 0.8. As the experimental protocols are consistent to those presented here, it is reasonable to assume that the parameter θ for starting our simulations at the day of co-infection should be <0.2. Thus, we consider *M*(*t*_*c*_) = 0.1, where *t*_*c*_ is the moment of co-infection.

### Statistical Analysis

Graph Pad Prism 5.0 (Graph Pad software, La Jolla, USA) was used to perform the indicated statistical tests.

## Data Availability

The raw data supporting the conclusions of this manuscript will be made available by the authors, without undue reservation, to any qualified researcher.

## Ethics Statement

Niedersächsisches Landesamt für Verbraucherschutz und Lebensmittelsicherheit, animal permit code: 16/2319.

## Author Contributions

NS-C, SS-K, HC, JB, and OK performed the experiments. All authors discussed and wrote the paper. EH-V performed the modeling analysis. DB and EH-V supervised the project.

### Conflict of Interest Statement

The authors declare that the research was conducted in the absence of any commercial or financial relationships that could be construed as a potential conflict of interest.
