# Supplementary material for: In vivo Neutralization of Pro-inflammatory Cytokines During Secondary Streptococcus pneumoniae Infection Post Influenza A Virus Infection
Source: Front Immunol. 2019 Aug 14;10:1864. doi: 10.3389/fimmu.2019.01864 (PMC6702285; doi:10.3389/fimmu.2019.01864)
Supplement: Supplementary file 1 [file Data_Sheet_1.PDF]

Supplementary Material

In vivo neutralization of pro-inflammatory cytokines during secondary *Streptococcus pneumoniae* infection post influenza A virus infection

Niharika Sharma-Chawla[1,2,3,+], Sabine Stegemann-Koniszewski[2,3,4,+], Henrike Christen[2], Julia D. Boehme[2,3], Olivia Kershaw[5], Jens Schreiber[4], Carlos A. Guzmán[6,7], Dunja Bruder[2,3, §], and Esteban A. Hernandez-Vargas[1, §,*]

1 Frankfurt Institute for Advanced Studies, Frankfurt am Main, Germany

2 Immune Regulation Group, Helmholtz Centre for Infection Research (HZI), Braunschweig, Germany

3 Infection Immunology Group, Institute of Medical Microbiology, Infection Prevention and Control, Health Campus Immunology, Infectiology and Inflammation, Otto-von-Guericke University Magdeburg, Germany

4 Experimental Pneumology, University Hospital of Pneumology, Health Campus Immunology, Infectiology and Inflammation, Otto-von-Guericke University Magdeburg, Germany

5 Department of Veterinary Medicine, Institute of Veterinary Pathology, Free University Berlin, Germany

6 Department of Vaccinology and Applied Microbiology, HZI, Braunschweig, Germany

7 Centre for Individualized Infection Medicine (CiiM), Hannover, Germany

[+] These authors contributed equally to this work

[§] These authors contributed equally to this work

* Corresponding Author: vargas@fias.uni-frankfurt.de

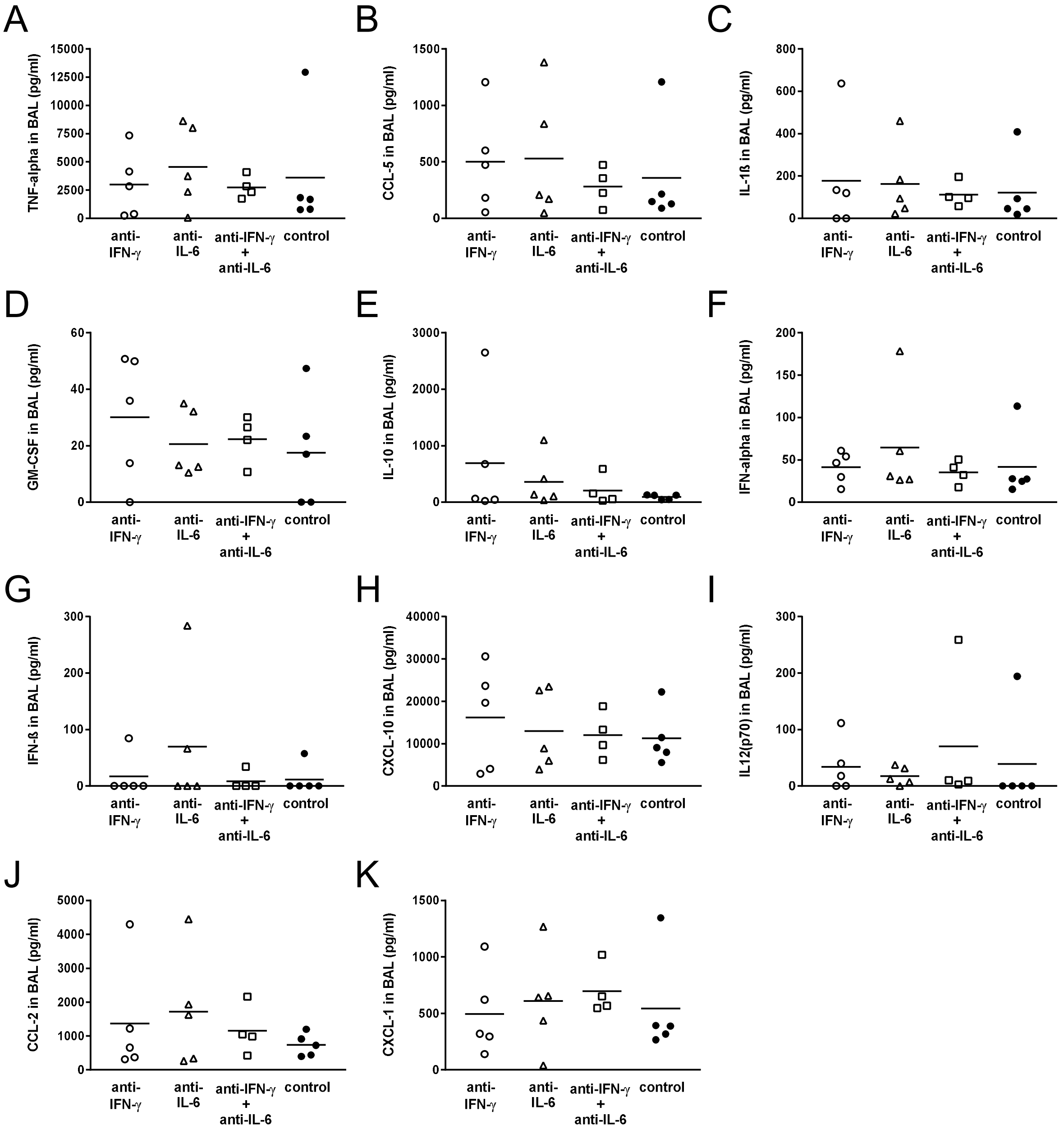

Supplementary Figure 1: Neutralization of IFN-γ alone, IL-6 alone and neutralization of IFN-γ and IL-6 during co-infection with 0.32 $TCID_{50}$ IAV and 1x10^{6} CFU *S. pn.* do not significantly affect airway TNF-α, CCL-5, IL-1ß, GM-CSF, IL-10, IFN-α, IFN-ß, CXCL-10, IL-12p70, CCL-2 and CXCL-1 levels. Mice were infected intranasally (i.n.) with 0.32 $TCID_{50}$ IAV on day 0. On day 5, mice were intraperitoneally (i.p.) injected with anti-IFN-γ antibody alone or in combination with anti-IL-6 antibody. On day 7, mice were treated with anti-IFN-γ antibody alone or in combination with anti-IL-6 antibody oropharyngeally (o.p.) and at the same time infected with 1x10^{6} CFU *S. pn.* o.p.. The control group was treated with an isotype IgG antibody and infected likewise. **A) - K)** Protein levels of TNF-α, CCL-5, IL-1ß, GM-CSF, IL-10, IFN-alpha, IFN-ß, CXCL-10, IL-12p70, CCL-2 and CXCL-1 in bronchoalveolar lavage (BAL) 24 h post o.p. antibody treatment and *S. pn.* infection. Data are shown for individual mice indicating the median/group.

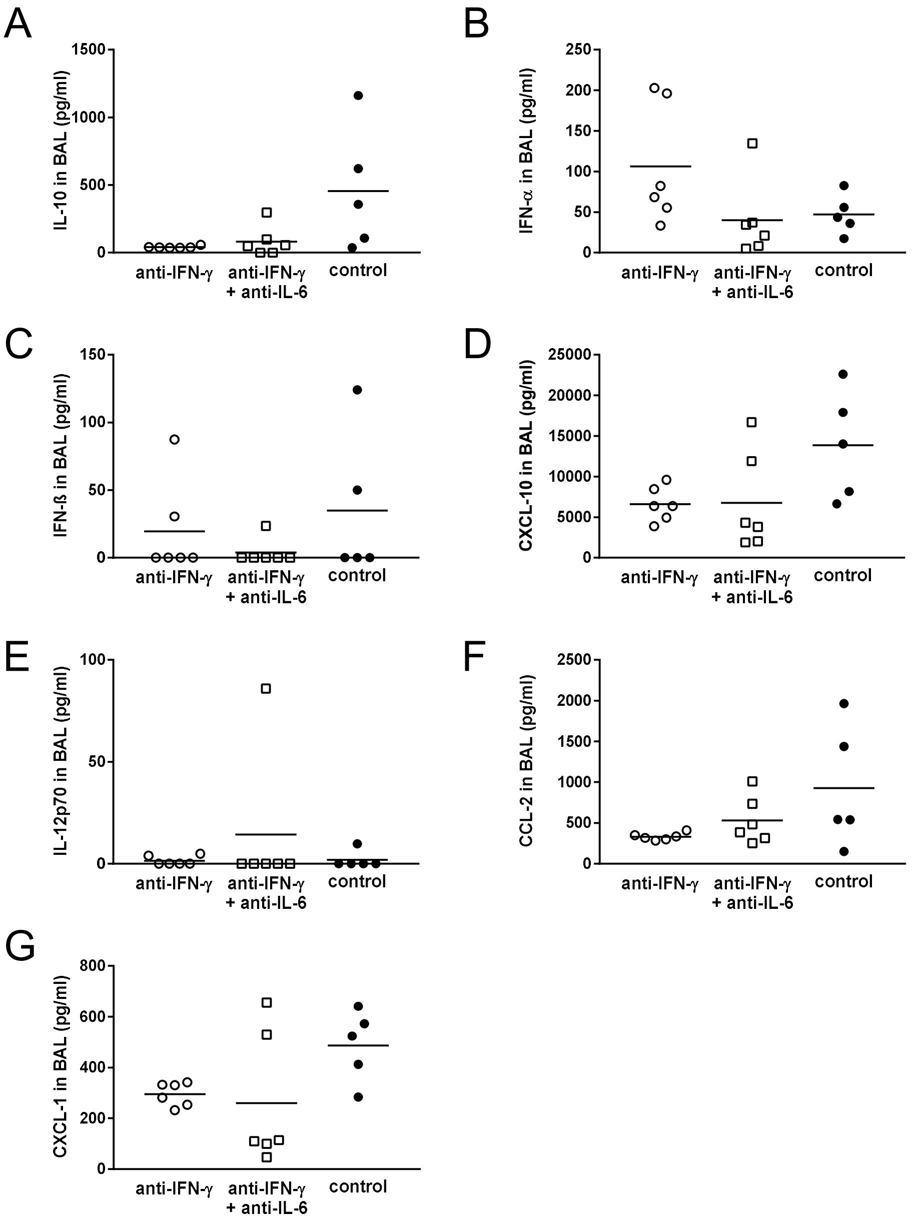

Supplementary Figure 2: Neutralization of IFN-γ and simultaneous neutralization of IFN-γ and IL-6 during co-infection with 0.17 $TCID_{50}$ IAV and $1x10^6$ CFU *S. pn.* do not significantly affect airway IL-10, IFN-α, IFN-ß, CXCL-10, IL-12p70, CCL-2 and CXCL-1 levels. Mice were infected intranasally (i.n.) with 0.17 $TCID_{50}$ IAV on day 0. On day 5, mice were intraperitoneally (i.p.) injected with anti-IFN-γ antibody alone or in combination with anti-IL-6 antibody. On day 7, mice were treated with anti-IFN-γ antibody alone or in combination with anti-IL-6 antibody oropharyngeally (o.p.) and at the same time infected with $1x10^6$ CFU *S. pn.* o.p. The control group was treated with an isotype IgG antibody and infected likewise. **A) - G)** Protein levels of IL-10, IFN-α, IFN-ß, CXCL-10, Il-12p70, CCL-2 and CXCL-1 in bronchoalveolar lavage (BAL) 24h post o.p. antibody treatment and *S. pn.* infection. Data are shown for individual animals indicating the median/group and are compiled from two independent experiments.

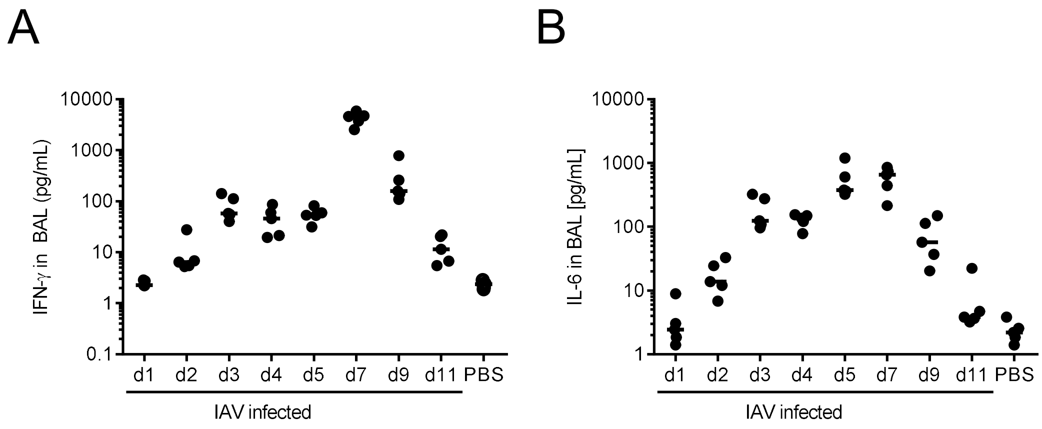

Supplementary Figure 3: Kinetics of respiratory IFN-γ and IL-6 following infection with 0.32 $TCiD_{50}$ IAV. Mice (n = 5) were infected intranasally with 0.32 $TCID_{50}$ IAV or treated with PBS. Mice were sacrificed at the indicated time points (day 1 for controls) and the concentrations of **A)** IFN-γ as well as **B)** IL-6 in bronchoalveolar lavage were assessed by a bead-based multiplex assay for flow cytometry. Data are shown for individual mice indicating the median/group.
